# Role of exosomes in the development of the immune microenvironment in hepatocellular carcinoma

**DOI:** 10.3389/fimmu.2023.1200201

**Published:** 2023-06-29

**Authors:** Tanghua Li, Jiapeng Jiao, Haoteng Ke, Wenshan Ouyang, Luobin Wang, Jin Pan, Xin Li

**Affiliations:** ^1^ The Second School of Clinical Medicine, Southern Medical University, Guangzhou, China; ^2^ The Department of Electronic Engineering, The Chinese University of Hong Kong, Hongkong, Hongkong SAR, China; ^3^ Zhujiang Hospital, Southern Medical University, Guangzhou, China

**Keywords:** exosome, hepatocellular carcinoma, tumor immune microenvironment, engineered exosome, immunotherapy

## Abstract

Despite numerous improved treatment methods used in recent years, hepatocellular carcinoma (HCC) is still a disease with a high mortality rate. Many recent studies have shown that immunotherapy has great potential for cancer treatment. Exosomes play a significant role in negatively regulating the immune system in HCC. Understanding how these exosomes play a role in innate and adaptive immunity in HCC can significantly improve the immunotherapeutic effects on HCC. Further, engineered exosomes can deliver different drugs and RNA molecules to regulate the immune microenvironment of HCC by regulating the aforementioned immune pathway, thereby significantly improving the mortality rate of HCC. This study aimed to declare the role of exosomes in the development of the immune microenvironment in HCC and list engineered exosomes that could be used for clinical transformation therapy. These findings might be beneficial for clinical patients.

## 1 Introduction

Hepatocellular carcinoma (HCC) accounts for 80% of liver cancer worldwide and is the main pathological type of primary liver cancer (1). Liver cancer is the second most common cause of cancer-related deaths worldwide, accounting for 8.3% of global cancer deaths. The number of new deaths caused by HCC is 830,180 (2). In China, HCC is the second leading cause of cancer-related deaths, lung cancer being the first (3). Although an increasing proportion of liver cancer is caused by alcoholic hepatitis and fatty liver disease, chronic hepatitis caused by the hepatitis B virus remains the leading cause of liver cancer worldwide (4, 5). Currently, the main treatment methods for HCC are surgical resection, liver transplantation, radiofrequency ablation, and vascular intervention (6, 7). However, early symptoms of patients with HCC are not obvious, resulting in delayed detection and diagnosis, leading to advanced stages and poor treatment outcomes (8). Immunotherapy serves as the most promising treatment for advanced HCC by activating the autoimmune system. Accumulating evidence shows that extracellular vesicles (EVs) play an essential role in developing the immune microenvironment in HCC, and engineered exosomes can effectively be used to regulate the immune microenvironment of liver cancer to achieve good therapeutic effects.

Tumor immune microenvironment (TIME) is the cellular environment for tumor survival, including cancer cells, innate and adaptive immune cells, stromal cells, endothelial cells, and cancer-related fibroblasts (9). Among these, immune cells play a dual role in cancer progression. The innate and adaptive immune systems in the TIME play a key role in the immune monitoring and regulation of cancer. However, a large number of immunosuppressive and inflammatory cells in the TIME can form an immunosuppressive microenvironment through intercellular communication, thus participating in the occurrence, development, and metastasis of cancer. Moreover, studies have shown many EVs in the microenvironment of HCC (10), which serve as the key media for intercellular communication and are closely related to the progression and metastasis of HCC. Exosomes are a subgroup of EVs (11). They are nano-sized vesicles with a lipid bilayer membrane and contain several bioactive molecules such as proteins and noncoding RNAs for intercellular communication to regulate the intercellular microenvironment and immune system (12, 13). Meanwhile, exosomes also play an essential role in promoting tumor invasion and metastasis. Tumor-derived exosomes have been proved to be vital in establishing pre-metastasis niches by creating an appropriate microenvironment at distant metastatic sites (14, 15). Exosomes are not easily cleared by the body due to their special properties, including low biological toxicity and immunogenicity, making them an excellent natural delivery system (16). Depending on the molecular transport capacity and targeting characteristics of exosomes, researchers have developed specific cell-targeted delivery tools based on engineered exosomes. Cell and tissue specificities are imparted to exosomes by modifying their surface molecules (17, 18), and the co-expression of protein/RNA transporters in the donor cells enables the selective packaging of specific protein/RNA molecules into the exosomes (19). Exosomes can also be used as cell-free vaccines for treating cancer and have shown encouraging results clinically (20). Therefore, using engineered exosomes to trigger antitumor immunity has become a promising treatment strategy (21).

This study aimed to review the role of secretions in regulating various immune cells in the liver to form an immunosuppressive microenvironment and participate in the occurrence, development, and metastasis of HCC, besides the clinical application of engineered exosomes. Also, the purpose was to understand further the mechanism of exosomes in mediating immunosuppression in the development of the immune microenvironment in HCC and to explore the therapeutic potential of engineered exosomes in HCC. The study aimed to provide new insights into the treatment of HCC in the future.

## 2 Role of exosomes in the inhibitory innate immune microenvironment of HCC

### 2.1 Macrophages

As an important part of the TIME, macrophages are mainly involved in HCC immune escape. They are also engaged in the invasion, proliferation, metastasis, and angiogenesis of HCC (22). They are plastic and heterogeneous because they are induced and polarized by stimuli from the surrounding environment as well as their own partial environment (23). The polarization of macrophages is determined by stimulus-inducing substances and subsequent gene expression patterns (24).

The polarization pathways of macrophages are grouped into the classical pathway (M1 macrophages) and the alternative pathway (M2 macrophages). In HCC, tumor-associated macrophages (TAMs) are important cellular components constituting the TIME (25). M1 macrophages are the major component of TAMs in early tumor emergence and progression (26). They are often characterized by high interleukin (IL)-12, low IL-10, and high CD86 expression (27).

During advanced tumor development, TAMs transform into M2 macrophages characterized by low IL-12 and high IL-10 expression (28). These TAMs mainly express products of M2 macrophages, such as the inflammatory suppressor IL-10, Th2 chemokine (C-C motif) ligand (CCL)-18 and CCL22, and CD206. Therefore, they are considered a subtype of M2 macrophages (27, 29). Chemokines, such as CCL3, CCL18, and CCL22 (macrophage-derived chemokines), and CXC-type chemokines, such as chemokine (C-X-C motif) ligand (CXCL)-9 and CXCL10, are involved in the recruitment of TAMs (30). Further, the colony-stimulating factor 1 (CSF-1) and vascular endothelial growth factor (VEGF) also promote the infiltration of TAMs in tumor tissues (24). Besides chemokines, colony-stimulating factor 1 (CSF-1) and VEGF have been shown to play a role in the infiltration of TAMs in HCC. CSF-1 is a potent stimulator in recruiting monocytes to tumors, which can differentiate into macrophages. In contrast, VEGF is known to promote angiogenesis and induce the polarization of TAMs toward the M2 phenotype. M1 macrophages promote tumor cell necrosis in tumors through tumor necrosis factor-alpha (TNF-α) expression, reactive oxygen species (ROS) oxidation, direct phagocytosis, and Th1 activation (31). In contrast, M2 macrophages mediate HCC immune escape (8). M2 macrophages also promote HCC angiogenesis and enhance tumor cell invasion (32, 33).

**Figure 1:**
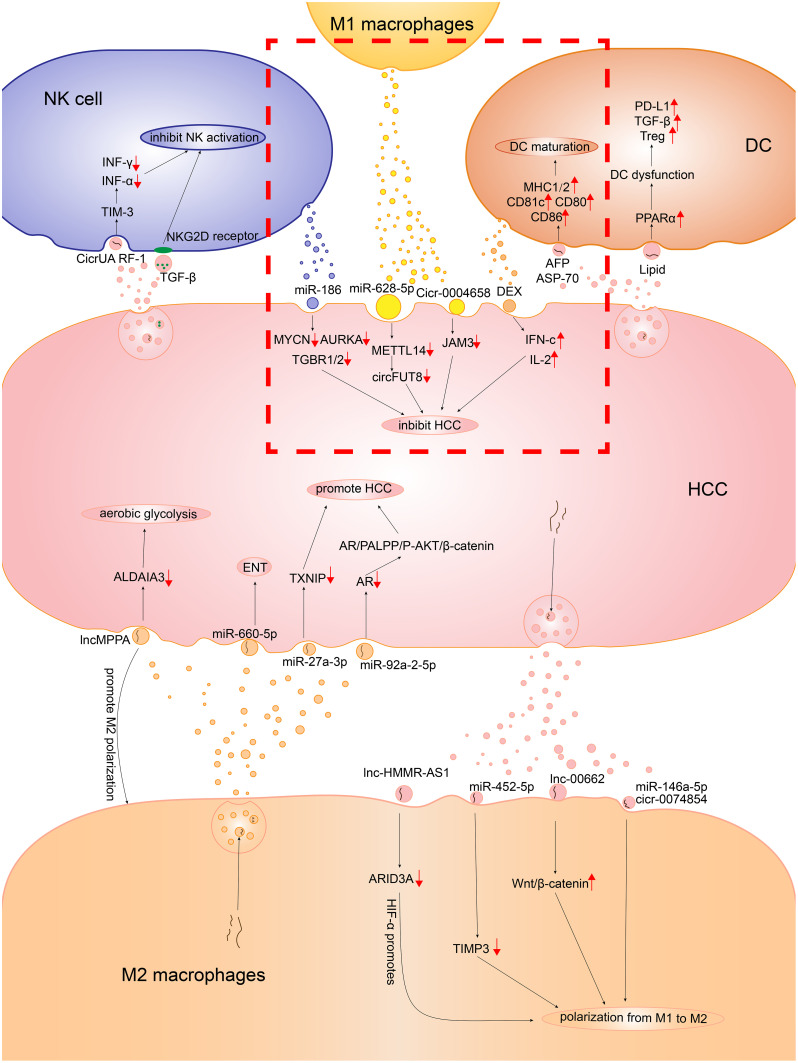
The detailed mechanism of the role exosomes played in the communication between HCC cells and the immune cells consisting of the innate immune microenvironment. In the red dotted box, there is the mechanism of how M1 macrophages, DCs, NKs inhibit HCC. The other part is the mechanism of how M2 macrophages, DCs, NKs promote HCC.

As an important component of intercellular communication, exosomes make a difference in the communication between HCC cells and macrophages. Macrophages mediate the immune surveillance of HCC via exosomes. M1 macrophage–derived exosomes can inhibit HCC progression (**Figure 1**). CircFUT8 was found high in the HCC, which is modified and facilitated by the human methyltransferase-like 14 (METTL14) expression. Exosomal miR-628-5p deprived from M1 macrophages were transfered into HCC cells to inhibit the expression of METTL14, so that M1 macrophages can suppress HCC progression (34). In addition, the exosomal hsa_circ_0004658 derived from recombination signal-binding protein-Jκ (RBPJ)-overexpressing M1 macrophages serve as the sponge for miR-499b-5p in HCC cells. They induce the downregulation of proto-oncogene junctional adhesion molecule 3 (JAM3) expression to inhibit HCC cell proliferation and promote apoptosis (35). Notably, the Notch pathway is an important pathway to regulate macrophage activation (36). The binding of Notch and RBPJ leads to the formation of a transcription factor that indirectly regulates the polarization of M1 macrophages (37). The decreased activation levels of the Notch pathway in M2 macrophages result in the activation of the RBPJ-mediated Notch pathway to increase the antitumor activity of M1 macrophages (23). However, HCC cells can also evade the antitumor effect by mediating the ferroptosis of M1 macrophages through exosomes. The number of M1 macrophages decreases in hepatitis B virus (HBV)-HCC tissues. It was shown that HCC cell–derived exosomal miR-142-3p promoted ferroptosis in M1 macrophages by upregulating the expression of solute carrier family 3 member 2 (SLC3A2), which promotes the proliferation of HCC (38). As HBV is a high-risk factor and an important cause of HCC, the findings of this study might have important implications for elucidating and exploring the mechanism of HBV infection causing HCC.

Although HCC cells evade the antitumor effect via the exosome-mediated ferroptosis of M1 macrophages, the immune escape of HCC cells is more likely to lead to the dominance of TAMs by M2 macrophages through regulating M2 polarization (**Figure 1**). Wang et al. co-cultured the PKH26-labeled HCC-derived exosomes and unpolarized macrophage human monocytic leukemia cells (THP-1) for 24 h. Subsequently, they detected PKH26 fluorescent dye in THP-1 (27), implying that HCC-derived exosomes could be taken up by macrophages. After Wang et al. co-incubated THP-1 and HepG2 HCC cells, the macrophages exhibited a CD86_low_/CD206_high_ phenotype, which was present in M2 macrophages (27). Studies showed that the proportion of M2 macrophages increased significantly with the addition of HCC-derived exosomes, and the specific markers of M2 macrophages, such as CCL17, CCL22, and Arg-1, as well as the products of M2 macrophages such as IL-10, were upregulated (39, 40). The findings showed that HCC cells promoted the polarization of M2 macrophages through exosomes. Mechanistically, miRNAs in HCC cell–derived exosomes bound to target RNAs and prevented the production of RNA translation products, thereby blocking the signaling pathways that regulated macrophage polarization, allowing macrophages to polarize toward the M2 phenotype. Anti-oncogene target tissue inhibitor of metalloproteinases-3 (TIMP3) can inhibit M2 macrophage polarization and promote apoptosis in HCC cells. HCC cell–derived exosomal miR-452-5p promoted M2 macrophage polarization by downregulating TIMP3 in macrophages (40–42). In addition, the translation factor sal-like protein-4 (SALL4) of HCC binds to the promoter of miR-146a-5p and regulates the derivation of exosomal miR-146a-5p (39). Exosomal miR-146a-5p promotes the polarization of M2 macrophages and suppresses the expression of interferon (IFN)-γ. IFN-γ inhibits the chemotaxis and polarization of M2 macrophages (43). Besides miRNAs, the exosomal contents of HCC include lncRNAs and circRNAs, which act as miRNA sponges to release the inhibition of miRNAs on target genes (44). LncRNA LINC00662 acts as a competitive endogenous RNA (ceRNA) binding to miR-15a, miR-16, and miR-107 to upregulate Wnt family member 3A (WNT3A) expression and secretion. As the proto-oncogene, WNT3A is associated with promotion of M2 macrophages. The HCC-derived exosomal LINC00662 activates Wnt/β-catenin signaling in macrophages and induces M2 macrophage polarization by stimulating the secretory effect of WNT3A (45). Another study showed that HCC cell–derived lncRNA hyaluronan–mediated motility receptor antisense RNA 1 (HMMR-AS1) bound competitively to miR-147a and inhibited the degradation of AT-rich interaction domain 3A (ARID3A). Exosomes carrying HMMR-AS1 can promote M2 macrophage polarization through this pathway, further accelerating HCC progression. Further, the hypoxia-inducible factor (HIF)-1α binds to the HMMR-AS1 promoter and promotes HMMR-AS1 transcription in a hypoxic environment, increasing the derivation of HCC exosomes (46). Wang et al. found that circRNA hsa_circ_0074854 knockdown exosomes could be delivered to macrophages to inhibit M2 macrophage polarization. Hence, HCC cell–derived exosomal hsa_circ_0074854 can promote M2 polarization. Moreover, Wang et al. found that hsa_circ_0074854 knockdown exosomes inhibited epithelial–mesenchymal transition (EMT) by reducing the stability of human antigen R (HuR) protein (27).

HCC-derived exosomes promote the transition of TAMs from M1 to M2, providing a favorable TIME for HCC development, invasion, and angiogenesis (**Figure 1**) (32). Before tumor angiogenesis takes place, hypoxic areas exist in HCC that produce HIF-1α. This HIF-1α then induces macrophage chemokines and promotes TAM infiltration (33, 47). Pieces of evidence suggest that TAM infiltration is the switch for tumor angiogenesis (48). The M2 phenotypes of TAM extensively aggregate in hypoxic avascular regions of tumors and produce VEGF and transforming growth factor beta (TGF-β), promoting tumor-associated angiogenesis (49–51). In hypoxic areas, TAMs can sense hypoxia and release VEGF; HIF-1α also upregulates VEGF expression in TAMs (52). Therefore, after M2 polarization, TAMs promote angiogenesis in HCC by secreting pro-angiogenic factors and cytokines, promoting tumor survival and progression. The prerequisite is the presence of HCC exosomes mediating the polarization of M2 macrophages. Besides promoting angiogenesis, M2 macrophages also promote the invasion and metastasis of HCC through the exosomal pathway. EMT is important in the development and metastasis of HCC (13). In the HCC cells treated with M2 macrophage–derived exosomes, the expression of epithelial marker E-cadherin decreased and the expression of mesenchymal markers N-cadherin and vimentin increased, which prompted the EMT of HCC cells. This result could be reversed by the miR-660-5p inhibitor. This suggested that M2 macrophage–derived exosomes harboring miR-660-5p promoted EMT in HCC. The co-culture of miR-660-5p-modified M2 macrophage–derived exosomes with HepG2 cells resulted in a significant increase in miR-660-5p expression and a decrease in Kruppel-like factor 3 (KLF3) expression in HCC cells (53). KLF3 is one of the indicators of cancer prognosis and tumor metastasis. The decrease in KLF3 expression promotes cancer development and stimulates cell metastasis (54). Similarly, M2 macrophage–derived exosomal miR-27a-3p can upregulate miR-27a-3p expression in HCC cells and induce HCC cell stemness through downregulating thioredoxin-interacting protein (TXNIP) to promote tumor cell proliferation. The upregulation of anti-oncogene TXNIP can eliminate the effect of miR-27a-3p on the CD133 positivity rate as well as SRY-Box transcription factor 2 (SOX2) and octamer-binding transcription factor-4 (OCT-4) expression in HCC, which are associated with cell stemness (50). Moreover, a previous study showed that miR-27a-3p expression was associated with tumor metastasis and angiogenesis in HCC (55). Besides, the incidence of HCC was higher in men than in women, probably because the development of HCC was regulated by sex hormones (56). The upregulation of the androgen receptor (AR) suppresses HCC development (57). However, M2 macrophage–derived exosomes are transferred into HCC cells, carrying miR-92a-2-5p that targets AR mRNA and inhibits AR translation. This promotes the development of HCC by affecting the AR/PH domain leucine-rich repeat protein phosphatase (PHLPP)/phospho-phosphatidylinositol 3-kinase-protein kinase B (p-Akt)/β-catenin signaling pathway (58). The aforementioned studies showed that M2 macrophage–derived exosomes downregulated the expression of anti-oncogenes and promoted the development of HCC. Hepatitis B is a risk factor for HCC. It is because hepatitis B e antigen (HBeAg) increases the MAPKAPK5-AS1 (MAAS) level of macrophages and promotes the polarization of M2 macrophages (59). The elevated MAAS is transferred from M2 macrophages to HBV-associated HCC cells via exosomes, targeting the proto-oncogene c-Myc and promoting tumor cell proliferation (59). Matrix metalloproteinases (MMPs) can degrade the extracellular matrix (ECM) in the early stages of many malignant tumors, playing an important role in the invasive potential and metastasis of tumors (60). He et al. found that the exosomes derived from HCC contributed to the increase of MMP-2 and MMP-9 through PI3K/AKT pathway (60). Notably, the elastin fragments produced by macrophage-derived MMP-9 promote monocyte chemotaxis (61). Moreover, MMP-9 can decompose ECM and release VEGF to accelerate tumor angiogenesis (62, 63). Xu et al. found that M2 macrophage–derived exosomal lncMMPA promoted the polarization of M2 macrophages and acted as a sponge for miRNA-548, which was transferred into HCC cells to promote the transcription of ALDH1A3. This promoted aerobic glycolysis in HCC cells and could have implications in the progression of HCC (64). The metabolic reprogramming of HCC, which is the aerobic glycolysis in HCC cells, is a common feature of HCC. It can supply sufficient energy and macromolecules to cells and promote the rapid proliferation of tumor cells (65, 66). With the increase in the infiltration of TAM and M2 macrophage–derived exosomes, the expression of glycolytic enzymes glucose transporter protein 1 (GLUT1) and hexokinase 2 (HK2) is elevated in HCC cells, which are the key components of glycolysis in HCC tissues (64). Besides upregulating the expression of pro-oncogenes, promoting biogenesis, and increasing glycolysis, the mechanisms by which M2 macrophages affect HCC cell development through exosomes have not been fully explored and need further investigation.

### 2.2 Dendritic cells

Dendritic cells (DCs) originate from hematopoietic stem cells in the bone marrow and act as antigen-presenting cells in the immune system, stimulating antitumor immune responses (**Figure 1**) (67). Studies have demonstrated that HCC tumor–derived exosomes (TEXs) can serve as the antigen sources for DC-mediated antitumor immunity. The TEXs of Hepa1-6 HCC cells contain AFP, glypican 3 (GPC3), and heat-shock protein 70. After the co-culture of TEXs and DCs for 24 h, the level of DC marker CD11c increases. It shows that TEXs can stimulate the maturation and differentiation of DCs. Moreover, the expression of major histocompatibility complex class (MHC) I, MHC II, co-stimulatory factors CD80 and CD86, and intercellular adhesion molecules increases in the co-cultured cells. It shows that DCs stimulated by TEXs can differentiate and mature normally, and have the ability to stimulate T cell maturation (68). TEXs carrying tumor cell dsDNA stimulate DCs and elicit antitumor immunity. Radiotherapy increases the accumulation of dsDNA in breast cancer cells and promotes the transfer of dsDNA into DCs by releasing exosomes. Besides upregulating co-stimulatory factors, DCs stimulated by dsDNA also activate the GMP–AMP synthase/stimulator of interferon genes (cGAS/STING) pathway to increase the expression of IFN-I. A previous study showed that IFN-I enhanced DC-mediated cross-presentation of tumor antigens to cytotoxic T lymphocytes (CTLs) (69). The cGAS/STING pathway is also widely present in the TIME of HCC (70, 71). Therefore, it is hypothesized that DCs can phagocytose TEX-carrying dsDNA in HCC, which activates the cGAS/STING pathway to exert antitumor immunity. DC-derived exosomes (DEXs) can also cause tumor regression in an HCC mouse model. The TIME of mice is significantly improved after treatment with AFP-carrying DEXs. The levels of IFN-c and IL-2 increase and the levels of IL-10 and TGF-β decrease, with an influx of CD8^+^ T cells (6). Moreover, the presence of MHC I and MHC II and co-stimulatory molecules on the surface enable DEXs to promote the antitumor effects of T cells and natural killer cells. Despite the stimulatory effect of HCC-derived exosomes on DCs, the immunosuppressive effects of HCC exosomes in inhibiting DC differentiation and inducing tolerogenic DCs in the local TIME should not be ignored. Exosomes from HCC are abundant in glycolipids, fatty acids, and phosphatidylserine (72). Peroxisome proliferator–activated receptor α (PPARα) expression is upregulated when DCs engulf lipid-rich exosomes, which is a major regulator involved in lipid, carbohydrate, and amino acid metabolism. PPARα leads to excess lipid droplet biogenesis, causing an increase in intracellular lipid mass and subsequently enhanced fatty acid oxidation, which in turn induces DC immune dysfunction (73). Immune-dysregulated DCs significantly upregulate the expression of inhibitory checkpoint proteins such as programmed death-ligand 1 (PD-L1) and signal-regulatory protein α (SIRPα) and increase the expression of immunosuppressive factor TGF-β. Moreover, immune-dysfunctional DCs can directly suppress the proliferation of CD8^+^ T cells and induce regulatory T lymphocyte (Treg) production (73).

Besides the immune-dysfunctional DCs induced by HCC-derived exosomes, the cells in the TIME can also modulate the function of DCs via exosomes. HCC-associated fibroblasts (hCAFs) have robust DC recruitment capacity (**Figure 1**). However, hCAF-derived IL-6 mediates the activation of the activate transcriptional activator 3 (STAT3) pathway in DCs and subsequent upregulation of indoleamine 2,3-dioxygenase (IDO), leading to the impaired function of T cells and the promotion of Tregs (74). The co-culture of breast cancer cell–derived exosomes with myeloid precursor cells promotes IL-6 release and activates the STAT3 pathway in myeloid precursor cells, inhibiting the differentiation and maturation of myeloid precursor cells to DCs (67). Therefore, it is hypothesized that HCC cells and hCAFs also activate the IL-6/STAT3 pathway via derived exosomes, thereby inhibiting DC maturation and impairing DC function. Further, the TIME has Tregs that suppress antitumor immunity. Treg-miRNAs, such as miR-150-5p and miR-142-3p, are transferred to DCs via derived exosomes. After Treg miRNAs are transferred with DCs, the release of IL-10 increases and the suppression of antitumor responses is enhanced (75). Moreover, miR-150-5p regulates IL-10 expression in human CD4^+^ T cells. The overexpression of miR-142-3p in DCs is also associated with reduced T cell activation (76, 77). The aforementioned evidence further confirms the feasibility of Tregs regulating DCs through exosomes.

### 2.3 Natural killer cells

Natural killer (NK) cells are prototype innate lymphoid cells with powerful cell-killing functions, which mediate antitumor and antiviral responses (**Figure 1**) (78). Exosomes from NK cells can also play a role in killing tumor cells by transporting miR-186 (79). NK cells distinguish the “self” from “missing-self” or “non-self” mainly through surface activating and inhibitory receptors, and regulate their activity through receptor signal transduction (80). Nevertheless, the tumor cells restrain the function of NK cells via multiple mechanisms and escape the NK cell cytotoxicity (**Figure 1**).

Natural killer group 2D (NKG2D) is the most representative activating receptor for NK cells, which recognizes MHC I chain–related molecules A and B and a family of six cytomegalovirus UL16-binding proteins (ULBP1–6). Normal cells express a limited number of NKG2D ligands on the surface, which are highly expressed in tumor cells. Besides, NKG2D ligands engage NKG2D to activate NK cells and exert cytolytic function (81). Unexpectedly, a large number of studies have revealed a negative correlation between tumor-derived exosomes and the level of NK cell–activating receptor NKG2D. The co-incubation of melanoma cell–derived exosomes, human acute myeloid leukemia blast–derived exosomes, and head and neck cancer–derived exosomes with NK cells significantly downregulated NKG2D expression (82–84). The expression of soluble NKG2D ligands (MICA, MICB, and ULBP1-3) on the surface of tumor exosomes and the delivery of TGF-β1 to NK cells are factors driving the downregulation of NKG2D. The downregulation of NKG2D was completely recovered after neutralizing TGF-β1 (85). Likewise, the serum levels of TGF β1 and MICA in HCC were higher than those in the control group (86, 87). MICA and MICB were also expressed in HCC-derived exosomes (88). It suggested that HCC-derived exosomes might downregulate NKG2D expression on the surface of NK cells and block cell activation via NKG2D ligands and TGF-β1, thus damaging the function of NK cells.

HCC-derived exosomes accelerate tumor growth by restraining the cytotoxicity of NK cells. After treatment with IL-6, miR-143-3p expression in HCC-derived exosomes increased and was negatively correlated with NK cells (89). HCC-derived exosomes overexpressed circUHRF1, which was associated with an attenuated proportion of NK cells and tumor infiltration. CircUHRF1 inhibited the secretion of IFN-γ and TNF-α by NK cells by enhancing the expression of TIM-3 via the degradation of miR-449c-5 (90). Meanwhile, miR-92b is also overexpressed in the exosomes from HCC. The exosomal miR-92b from HCC is transferred to NK cells, resulting in cytotoxicity mediated by CD69 and NK cell degradation (91).

## 3 Role of exosomes in the inhibitory adaptive immune microenvironment in HCC

Many antigens are present in the blood that the liver receives from the intestines. The liver has developed immune tolerance mechanisms over a long period of evolution to prevent autoimmune injury. Further, most patients with HCC have a history of chronic hepatitis and inflammation, resulting in a large number of immunosuppressive cells in the liver (92). These immune cells are essential in the immune microenvironment of the liver, which in turn promotes the occurrence and development of HCC (9). Recent studies have shown the vital role of exosomes in the formation of an immunosuppressive microenvironment in HCC (13). Exosomes may influence lymphocytes in the TIME to play a role in immunosuppression, tumor progression, and metastasis. The mechanisms by which exosomes regulate T and B lymphocytes in HCC are as follows.

### 3.1 T lymphocytes

T lymphocytes comprise nearly half the total lymphocytes in a healthy liver (93). Furthermore, they play a significant role in the immune responses of HCC (94). CD8^+^ T lymphocytes and CD4^+^ T lymphocytes are major players in the immune response. CD8^+^ CTLs infiltrating the tumor specifically inhibit tumor growth, patrolling the liver and killing tumor cells through cytotoxicity (95, 96). Meanwhile, CD4^+^ T lymphocytes maintain the function of other immune cells (97).

The development of HCC is a complex multi-step process. Genetic and epigenetic differences exist in normal cells before they develop into cancer cells (98). Theoretically, the immune system recognizes these differences and activates T lymphocytes to eliminate cancer cells. However, a wide range of tumor cells evades the surveillance of immune cells (99). This seems to be related to the large number of exosomes present in HCC (10). Moreover, cancer cells usually produce more exosomes than normal cells, and tumor-derived exosomes have a strong capacity to remodel the TIME (100). Mounting evidence suggests that exosomes derived from tumor cells inhibit the immune function of T lymphocytes. This includes decreasing the cytotoxic activity of CD8^+^ T lymphocytes, inhibiting their proliferation and promoting their apoptosis, and inhibiting the function of CD4^+^ T lymphocytes, leading to Th17/Treg imbalance (8, 101–103)

**Figure 2:**
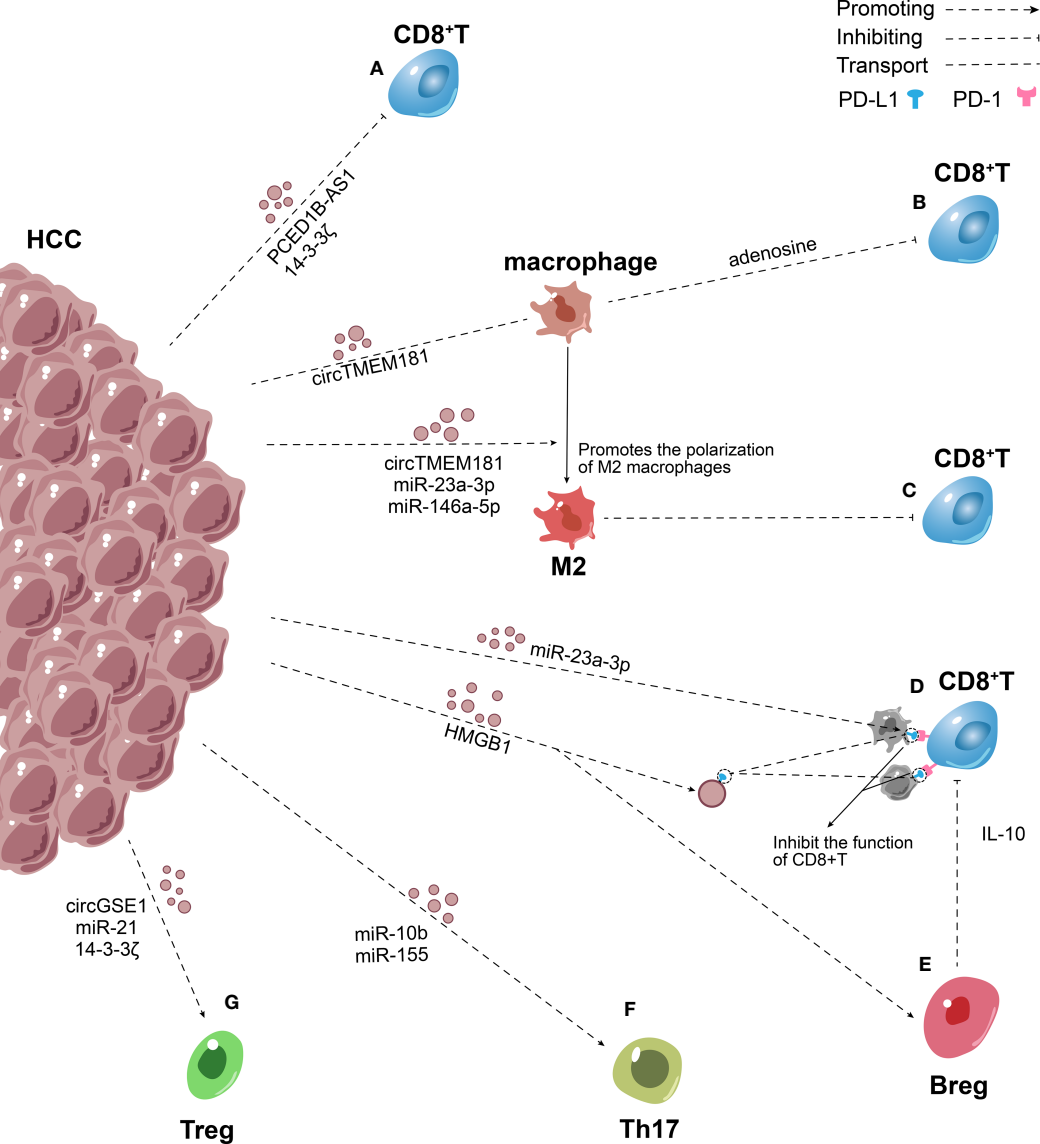
Role of exosomes in the inhibitory adaptive immune microenvironment in HCC. **(A)** HCC secrete PCED1B-AS1, 14-3-3ζ rich exosomes to inhibit the function of CD8^+^ T lymphocytes directly. **(B)** HCC transmitted overexpressed circTMEM181 to macrophages through exosomes, leading to the enrichment of adenosine in the TIME and thereby damaging the function of CD8^+^ T cells. **(C)** Exosomes containing circTMEM181, miR-23a-3p, miR-146a-5p secreted by HCC promote M2 macrophage polarization, thus impairs the CD8^+^ T cells. **(D)** HMGB1 mRNA promotes the production of PD-L1-rich exosomes, exosomes transport PD-L1 to macrophages and other HCC cells. PD-L1 on the surface of tumor-derived exosomes and cells interact with PD-1 on the surface of CD8^+^ T cells to induce apoptosis, exhaustion, and inactivation of CD8^+^ T cells. **(E–G)** HCC–derived exosomes increased Breg, Treg and Th17 expression via many mechanisms and mediated the imbalance of Th17/Treg.

High infiltration levels of CD8^+^ T lymphocytes predicted higher survival rates for HCC (104). CD8^+^ T lymphocytes recognize and specifically kill tumor cells. Nevertheless, a large percentage of CD8^+^ T lymphocyte phenotypes are altered in HCC, expressing high levels of programmed death-1 (PD-1). The proliferation and activity of these cells decrease through the PD-1/PD-L1 pathway, thus suppressing the killing of tumor cells (105). Chen et al. found that metastatic melanomas released exosomes carrying PD-L1 on their surface into the TIME and circulation. These exosomes mainly targeted CD8^+^ T lymphocytes, inhibited the function of CD8^+^ T lymphocytes, and promoted tumor growth. The expression of PD-L1 on the surface of exosomes was upregulated by IFN-γ (106). PD-L1 on the surface of tumor-derived exosomes may interact with PD-1 on the surface of CD8^+^ T lymphocytes to transmit inhibitory signals, induce apoptosis, exhaustion, and inactivation of CD8^+^ T lymphocytes, and inhibit the production of IL-2 and IFN-γ (107). Low expression of IL-2 and IFN-γ is a sign of T cell depletion (108, 109). However, whether HCC can secrete PD-L1-rich exosomes to inhibit the immune function of CD8^+^ T cells, thus mediating tumor escape, remains unclear. Wei et al. reported that hepatocytes overexpressing high–mobility group protein 1 gene (HMGB1)/rapamycin-insensitive companion of mTOR (RICTOR) 3’-UTR released more PD-L1-rich exosomes than normal hepatocytes. HMGB1 mRNA is highly expressed in HCC and functions as a microRNA sponge to competitively bind to the miR-200 family (especially miR-429) to promote the expression of RICTOR mRNA. The RNA–RNA crosstalk network upregulates the PD-L1 expression of HCC and promotes the production of PD-L1-rich exosomes by activating the mTORC1–P70S6K pathway (110). The PD-L1 on the tumor-derived exosomes not only binds to the PD-1 on CD8^+^ T cells, but is transferred to other cells. Emerging evidence indicates that exosomes transport PD-L1 from PD-L1-positive to PD-L1-negative breast cancer cells, thus inhibiting the activities of T cells (**Figure 2**) (111). Despite no direct evidence that PD-L1-positive HCC cells transport PD-L1 to PD-L1-negative HCC cells, it is may be a potential mechanism for way of HCC immune escape.

Additionally, Wang et al. indicated that 14-3-3 ζ was transferred from HCC to CD8^+^T cells, leading to T cell exhaustion (101). Besides transferring proteins, tumor-derived exosomes transfer genetic material to target cells to inhibit T cell immune function (12). The expression of PCED1B-AS1 is upregulated in HCC, then PCED1B-AS1 assembly into exosomes and release into the TIME and circulation. Further, other HCC cells take up these exosomes. PCED1B-AS1 acts as a microRNA sponge to inhibit mir-194-5p expression and enhance the expression of PD-L1 and PD-L2, thus damaging the activity of T lymphocytes and macrophages simultaneously (107, 112). Furthermore, most studies showed that exosomes released by HCC contained high levels of miR-222, which has been verified to downregulate intercellular cell adhesion molecule-1 (ICAM-1) on tumor cells, thereby inhibiting the tumor-killing effect of CTLs (**Figure 2**) (113–115). This immunological suppression mediated by HCC-derived exosomes may be a potential mechanism of tumor escape.

Also, Zhang et al. showed that HCC produced exosomes containing miR-15a-5p, which directly targeted CD8^+^ T cells and inhibited the expression of PD-1 on CD8^+^ T cells, thus inhibiting cancer progression. However, cancer cell–derived exosomes carried fewer miR-15a-5p than normal cell–derived exosomes (116). Further, a recent study showed that exosomes excreted tumor-suppressive circRHOBTB3 out of colorectal cancer cells to promote tumor escape (117). This suggested that tumor-derived exosomes maintain the proliferation of tumor cells by reducing the growth-inhibiting RNA in tumor or immune cells. More attention should be given to growth-inhibiting miRNAs and developing new tumor treatments.

Exosomal miRNAs indirectly affect T cells by changing the phenotype of macrophages, which is a mechanism to mediate the immunosuppression of HCC (**Figure 2**) (118). Yin et al. demonstrated that HCC secreted exosomes containing miR-146a-5p, which were taken up by macrophages. This process activated the NF-κB pathway in macrophages and induced inflammatory factors, which in turn promoted M2 polarization. Macrophages treated with HCC-derived exosomes upregulated the expression of inhibitory receptors including T cell immunoglobulin and mucin domain-containing protein 3 (TIM-3), lymphocyte-activation gene 3 (LAG-3), cytotoxic T-lymphocyte-associated protein 4 (CTLA4), T cell immunoreceptor with Ig and ITIM domains (TIGIT) and PD-1 on T cell surface, downregulated the production of IFN-γ and TNF-α, and induced T cell exhaustion to cause immunosuppression. The exosomes enriched with miR-146a-5p were controlled by the transcription factor SALL4, which was highly expressed in HCC (39). These basic findings were consistent with previous findings showing that endoplasmic reticulum (ER) stress–related exosomes were involved in M2 macrophage polarization, promoted the apoptosis of CD8^+^ T cells, and reduced the production of IL-2 in T cells. Upon induction of ER stress signal, HCC cells were found to secrete exosomes containing miR-23a-3p that were taken up by macrophages, which in turn increased PD-L1 expression on macrophages. This effect was brought about by the downregulation of phosphatase and tensin homolog (PTEN) expression and upregulation of p-Akt expression. PD-L1 expressed by macrophages induced CD8^+^ T cell apoptosis (8). Moreover, a recent study showed that overexpressed golgi membrane protein 1 (GOLM1) in HCC stabilized PD-L1 via CSN5-mediated PD-L1 deubiquitination, and facilitates PD-L1 sorted into the exosomes by inhibiting Rab27b and then transferred to macrophages to inhibit the growth of CD8^+^ T cells (119). Furthermore, HCC cells and macrophages synergistically affected the activity of CD8^+^ T cells through the ATP–adenosine pathway. Recent studies indicated that HCC transmitted overexpressed circTMEM181 to macrophages through exosomes, elevating the expression of CD39 in macrophages. CD39 expression in macrophages and CD73 expression in HCC cells synergistically activated the ATP–adenosine pathway, leading to the enrichment of adenosine in the TIME and thereby damaging the function of CD8^+^ T cells (120). Promoting M2 polarization is an important mechanism via which HCC indirectly inhibits the growth of T cells. Tumor-derived exosomes not only influence the surface and microenvironment of macrophages but also promote M2 macrophage polarization (8, 39, 120). The infiltration of M2 macrophages in tumors impairs the CD8^+^ cytotoxic T cells through the R-spondin (Rspo)-leucine-rich repeat-containing G protein-coupled receptor 4 (Lgr4)/signal transducer and activator of transcription 3 (STAT3) axis.

Exosomes derived from other immune cells in HCC induce the depletion of CD8^+^ T cells, which is another mechanism of immune escape induced by HCC. Several studies reported that M2 macrophage–derived exosomes and Treg cell–derived exosomes suppressed the proliferation of CD8^+^ CTLs in the TIME. M2 macrophage–derived exosomes promoted the exhaustion of CD8^+^ T cells through the miR-21-5p/YOD1/YAP/β-catenin axis, and Treg cell–derived exosomes decreased the secretion of perforin and IFN- γ from CD8^+^ T cells in HCC (121, 122).

CD4^+^ T lymphocytes have various subtypes and exert an antitumor effect in HCC via releasing cytokines and activating CD8^+^ T lymphocytes (123). On the one hand, the exosomes overexpressing PD-L1 in HCC reduced the function of CD8^+^ T lymphocytes; on the other hand, they damaged the activity of CD4^+^ T cells. Gong et al. demonstrated that norcholic acid (NorCA) reduced the levels of farnesoid X receptor and protein tyrosine phosphatase (SHP) in HCC, thus elevating the level of PD-L1 on the surface of HCC cells and their exosomes and enhancing exosome secretion. Exosomes induced by NorCA significantly increased the expression of PD-1 and TIM3 in CD4^+^ T cells; subsequently, the function of CD4^+^ T lymphocytes was impaired, promoting the proliferation, migration, and invasion of HCC (102). Additionally, another mechanism underlying HCC immune escape is the imbalance of Th17/Tregs. Tregs play a crucial role in maintaining normal immune homeostasis and preventing the occurrence of autoimmune diseases by suppressing the immune response (124). They also suppress antitumor immunity and result in tumor immune escape. This mechanism is exploited by tumor cells, which facilitates their accumulation in the TIME over time (125). On the one hand, Th17 activates transcriptional activator STAT3 and then upregulates the genes that induce tumor angiogenesis, promoting tumor growth and metastasis (126). On the other hand, it limits tumor growth by secreting IL-17 and IFN-γ that increase the expression of cytotoxic lymphocytes (CTL) in the TIME (127). Th17 and Treg inhibit each other, and the changes in their levels are opposite. The balance of Th17/Treg is the guarantee of immune function. Th17/Treg imbalance is an important indicator of HCC progression (128).

MiR-155 can be transferred to other HCCs via exosomes, promoting their proliferation. MiR-155 targets PTEN and restrains its expression, which is controlled by HBV X protein (HBX) overexpression (103, 129). PTEN is a highly conserved protein tyrosine phosphatase that can dephosphorylate inositol lipids and proteins. The decrease in PTEN expression is strongly associated with Th17/Treg cell imbalance (130). A previous study confirmed that PTEN promoted Th17 differentiation by attenuating the production of IL-2 (131). Further, miR-155 overexpression promoted the differentiation of Th17 and increased Th17 expression by restraining the expression of gene suppressor of cytokine signaling 1 (SOCS1) (132). In short, HCC transferred miR-155 to the target HCC cells through exosomes, mediated the imbalance of Th17/Treg, caused immunosuppression, and augmented HCC infiltration and metastasis (**Figure 2**).

Recruiting a large number of Tregs into the TIME is another mechanism for HCC to induce immunosuppression (**Figure 2**). The acidic microenvironment increases exosomal miR-21 and miR-10b expression in HIF-1α- and/or HIF-2α-dependent manner (133). MiR-21 enhances the proliferation and recruitment of Tregs to the TIME, while reducing the proportion of Th17 (134). Further, miR-10b targets GATA-binding protein 3 (GATA3) and PTEN and suppresses their expression, thus increasing the proportion of Th1 and Th17 cells but diminishing the proportion of Th2 and Tregs cells (135). Indeed, the overexpression of miR-21 and miR-10b eventually led to the proliferation and metastasis of HCC cells (133). Furthermore, Huang et al. showed that HCC-derived exosomes transported circGSE1 to CD4^+^ T cells, which served as a microRNA sponge to miR-324‐5p. HCC-derived exosomes promoted the differentiation of CD4^+^ T cells into Treg via regulating the miR-324-5p/TGFBR1/Smad3 axis (136). From this standpoint, exosomal circGSE1 can be considered a biomarker of immunotherapy for HCC. According to a recent study, patients with high expression of exportin 1(XPO1), interferon γ-inducible protein 30(IFI30), F-box protein 16(FBXO16), and calmodulin 1 (CALM1) and low expression of MORC family CW-type zinc finger 3 (MORC3) had a higher proportion of Tregs and higher expression of immune checkpoint molecules (137). Additionally, Wang et al. emphasized that CD3^+^ immature T cells took up exosomes containing 14-3-3 ζ secreted by HCC and tended to differentiate into Tregs (101).

### 3.2 B lymphocytes

Compared with T lymphocytes, the data on the role of B lymphocytes in the immune response to HCC is limited. The percentage of B cells in the liver is low, but they play a significant role in tumors by producing immunoglobulins, presenting antigens, providing co-stimulatory signals, and releasing cytokines (138). However, regulatory B-cell (Breg) subsets restrain host immune response, induce immunosuppression in HCC, and play a tumor-promoting role, which is related to the advanced stage of the disease and poor prognosis (139).

HCC-derived exosomes also regulate the expression of Bregs. Ye et al. emphasized that TIM-1 Bregs were critical in HCC immune escape. They reported that the HMGB1 expression was significantly higher in exosomes derived from HCC than in exosomes derived from other cells. Moreover, tumor-derived exosomes expand TIM-1 Breg cells via the HMGB1–Toll-like receptor 2/4 (TLR2/4)–mitogen-activated protein kinase pathway. TIM-1 Bregs produce a large amount of IL-10, which can inhibit the proliferation of CD8^+^ T cells and the production of TNF-α and IFN-γ, thus inducing immunosuppression and prompting the progression of HCC (140). In fact, other subsets of Bregs infiltrating HCC promote tumor immune escape via other mechanisms. Xiao et al. identified a novel tumor-promoting PD-1(hi) B-cell subset in human HCC. Another study found that PD-1(hi) B cells combined with PD-L1 to produce a large amount of IL-10, which attenuated the proliferation and activity of CD8^+^ T lymphocytes and provided favorable conditions for the progression of HCC (141). Previous studies also indicated that esophageal squamous cell carcinoma–derived exosomes augmented the expression of PD-1 in B cells and induced the differentiation of PD-1(hi) B cells (142). Future studies should investigate the relationship between HCC-derived exosomes and PD-1(hi) B cells. In summary, HCC-derived exosomes mediate the differentiation of Bregs, induce immunosuppression, and accelerate the progression of HCC (**Figure 2**).

## 4 Role of exosomes in the inhibitory immune microenvironment of other components in HCC

### 4.1 Cancer-associated fibroblasts

Cancer-associated fibroblasts (CAFs) are one of the most crucial components in the TIME and have been proved to play a significant role in the occurrence and development of HCC (143). Multiple previous studies showed that CAFs released diverse regulatory factors and synthesized and remodeled extracellular matrix, which had a crucial role in boosting tumor proliferation, metastasis, angiogenesis, and drug resistance (144). Recently, the interaction between CAFs and TIME has been identified as another key factor in promoting tumor progression (145).

Increasing evidence has revealed that CAFs promote tumor proliferation by mediating immunosuppression. Recent studies have shown different subsets of CAFs in tumors. Costa et al. identified CAF-S1 subsets in breast cancer, which were associated with immunosuppression. By secreting CXCL12, CAF-S1 attracts CD4^+^CD25^+^T lymphocytes and keeps them on their surface. Further, CAF-S1 enhances the differentiation of T lymphocytes into CD25^++High^FOXP3^High^ cells via express CD73, dipeptidyl peptidase-4 (DPP4) and B7H3. Finally, it upregulates the capacity of regulatory Treg to inhibit effector T cells (146). Another subset suppresses the T cell–dependent antitumor immune response by secreting TGF-β to attenuate the proliferation of CD8^+^ T cells and promote the recruitment of CD4^+^CD25^+^ T cells (147, 148). Furthermore, the recruitment of CD4^+^ CD25^+^ Treg cells to CAFs also depends on CCL5 (149). Moreover, FAP^+^PDPN^+^ CAFs have been demonstrated to suppress T cell proliferation in a nitric oxide–dependent manner in breast cancer (150). In fact, CAFs expressed PD-L1 and PD-L2 bound to PD-1 receptors on T cells and then inhibited T cell activity (146, 151). Notably, CAFs are related to the drug resistance of tumors to anti-PD1 or anti-PD-L1 immunotherapy (152). In esophageal cancer, CAFs suppress the recruitment of CD8^+^ T cells by secreting high levels of IL-6 (153). In addition, TGF-β secreted by CAFs is also involved in immunosuppression in the TIME. TGF-β inhibits the cytotoxic activity of CD8^+^ T cells via direct inhibition of perforin, granzyme A (Gzm A), Gzm B, IFN γ, and Fas ligand (FasL) (153, 154). Goehrig et al. found that CAFs secreted βig-h3, which reduced the proliferation and activation of CD8^+^ T cells (155).

**Figure 3:**
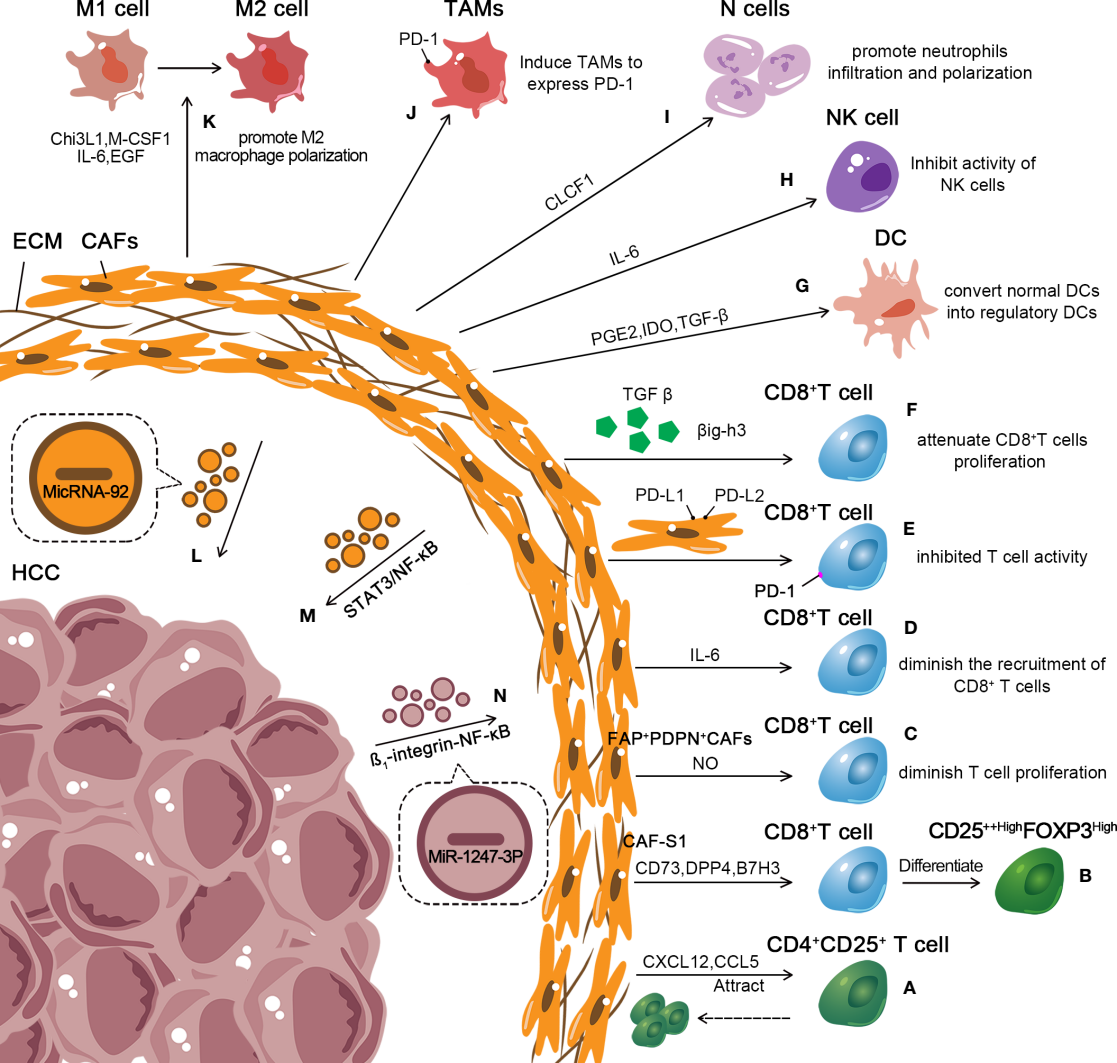
Role of exosomes in the inhibitory immune microenvironment of CAFs in HCC. **(A, B)** CAF-S1 enhances the differentiation of T lymphocytes into CD25^++High^FOXP3^High^ cells. Further, CAF-S1 attracts CD4^+^CD25^+^ T lymphocytes. And keeps them on their surface. **(C)** FAP+PDPN+ CAFs diminish T cell proliferation in a nitric oxide–dependent manner. **(D)** CAFs suppress the recruitment of CD8+ T cells by secreting high levels of IL-6. **(E, F)** CAFs secrete TGF-β, βig-h3 to attenuate the proliferation of CD8^+^ T cells and express PD-L1 and PD-L2 bound to PD-1 receptors on T cells to inhibit T cells activity. **(G–K)** CAFs affect the cytotoxic activity of DC and NK cells and enhance the recruitment and impact the polarization of TAMs and neutrophils, combine with promoting M2macrophage polarization. **(L–N)** CAF-derived exosomes containing microRNA-92 enhance the expression of PD-L1. CAF-derived exosomes promoted HCC resistance to cisplatin via STAT3/NF-κB pathway. HCC cell–derived MiR-1247-3p activated the β1-integrin-NF-κB signaling pathway in fibroblasts, converting NFs into CAFs.

Further, CAFs also restrain the innate immune response, enhancing the recruitment and polarization of TAMs and neutrophils and affecting the cytotoxic activity of DC and NK cells (156). Accumulating evidence suggests that CAFs secrete chitinase 3-like 1 (Chi3L1), M-CSF1, IL-6, and VEGF, which induce macrophage infiltration and promote M2 macrophage polarization (157, 158). Moreover, CSFs induce TAM to express PD-1, and PD-1 expression on the surface of TAMs leads to innate and adaptive antitumor immune response suppression (159). Song et al. observed CAFs in HCC-derived cardiotrophin-like cytokine factor 1 (CLCF1), which upregulated CXCL6 and TGF-β expression in HCC to promote neutrophil infiltration and polarization (160). In addition, CAFs derived from HCC produced a large amount of prostaglandin E2 (PGE2) and IDO to inhibit the activity of NK cells (161). TGF-β secreted by CAFs also inhibited the activity of NK cells by downregulating the expression of activating receptor NKG2D (162, 163). Cheng et al. proved that CAFs in HCC recruited DCs through IL-6-mediated STAT3 activation. CAFs converted normal DCs into regulatory DCs, which tended to express more immunosuppressive cytokines and induced Treg differentiation and T cell anergy (74). A large amount of ECM produced by CAFs forms a physical barrier, mitigates the migration of immune cells, and limits the attack of immune cells on the tumor, indirectly promoting immunosuppression (164). In summary, different subsets of CAFs in tumors impact adaptive and innate antitumor immune responses through various mechanisms, mediating drug resistance and boosting tumor growth (**Figure 3**).

A recent study demonstrated that tumor-derived exosomes play an important role in CAF transition (**Figure 3**). Mito et al. emphasized that normal fibroblasts (NFs) converted into CAFs after the uptake of tumor-derived exosomes in head and neck squamous cell carcinoma, suppressed T cell proliferation, and induced macrophage polarization (165). MiR-425-5p in breast cancer–derived exosomes induced the conversion of fibroblasts into CAFs via the TGF-β1/ROS signaling pathway (166). Similarly, HCC showed a strong ability to convert NFs into CAFs. MiR-1247-3p in highly metastatic HCC cell–derived exosomes targeted B4GALT3 and activated the β1-integrin-NF-κB signaling pathway in fibroblasts, converting NFs into CAFs (167). Further, HCC-derived exosomes secreted miRNA-21 into hepatic stellate cells (HSCs) and activated PDK1/Akt signaling pathways, prompting the transformation of HSCs into CAFs (168). CAFs are key in liver inflammation, fibrosis, and tumor progression. Further exploration of HCC-derived exosomes in activating CAFs may pave the way for new therapeutic strategies for HCC.

Besides secreting various cytokines, growth factors, and chemokines, CAFs also induce immunosuppression by deriving exosomes. Dou et al. found that the level of microRNA-92 isolated from CAF-derived exosomes increased significantly. Moreover, breast cancer cells treated with CAF-derived exosomes enhanced the expression of PD-L1 by augmenting the nuclear translocation of Yes-associated protein 1(YAP1) (169). HCCmiR-92a exhibited higher expression levels than those in normal tissues (170, 171). Furthermore, CAF-derived exosomes boosted circZFR expression in HCC cells via the STAT3/NF-κB pathway and promoted HCC resistance to cisplatin (**Figure 3**) (172). This provided a new target for treating HCC. However, in HCC, the relationship between CAF-derived exosomes and antitumor immune response has not yet been fully investigated. Hence, further understanding of the underlying mechanism is essential to better inform treatment decisions.

### 4.2 Hepatic stellate cells

HSCs are resident nonmesenchymal cells and major producers of ECM with fibroblastic features (173). An injury to hepatocytes stimulates the activation of HSCs, which marks the transformation of HSCs into myofibroblasts (174). In chronic liver disease, the imbalance between pro-fibrotic and anti-fibrotic effects leads to persistent activation, proliferation, and migration of HSCs. Subsequently, excessive production and deposition of ECM occur in the liver, leading to liver fibrosis (175). Damaged hepatocytes can activate HSCs through exosomes to cause liver fibrosis. HSCs can also activate HSCs and promote the development of liver fibrosis through autophagy and exosomes (176).

Acute viral hepatitis caused by viral infection is the main cause of liver fibrosis. The upregulation of miR-222 expression is found in HBV-infected hepatocytes. Further research confirmed that exosomal miR-222 secreted by HBV-infected hepatocytes could target and regulate the expression of the transferrin receptor, thereby inhibiting the ferroptosis of HSC LX2 and upregulating the activity of HSCs (177). Hepatitis C virus induces hepatocytes to secrete miR-1273g-3p-containing exosomes to downregulate PTEN, a gene that inhibits liver fibrosis, thereby increasing HSC activation (178). Metabolism-related fatty liver disease is also an important factor in liver fibrosis. The overexpression of miR-1297 in lipotoxic hepatocyte–derived exosomes promotes the proliferation and activation of HSCs through the PTEN/phosphoinositide 3-kinase (PI3K)/protein kinase B (ATK) pathway. After receiving lipotoxic hepatocyte–derived exosomes, the production of hydroxyproline, the expression of the activation marker α-smooth muscle actin (α-SMA), and the proliferation marker proliferating cell nuclear antigen (PCNA) increase in the LX2. The same effect can also be obtained by knocking out the PTEN of HSCs (179). Connective tissue growth factor 2 (CCN2) is an important component driving the activation of HSCs, and its regulation is influenced by HSC-derived exosomes. HSCs isolated in chronic ethanol-feeding or carbon tetrachloride (CCl4)-exposed mouse hepatitis models had high CCN2 expression and low miR-214 expression. However, HSCs isolated from normal mouse liver had low CCN2 expression and high miR-214 expression. CCN2 3′-UTR recipient-activated HSCs were transfected with wild-type CCN2 3′-UTR luciferase reporter and co-cultured with donor-activated HSCs transfected with pre-mir-214. The test result could be reversed using mutated CCN2 3’-UTR or exosome inhibitor GW4869. This demonstrated that miR-214 was indeed derived from HSCs and downregulated the CCN2 expression in HSCs (180).

Besides their involvement in liver fibrosis, HSCs are an important source of CAFs participating in the TIME (181). HSCs participate in the occurrence, development, and metastasis of tumors and secrete cytokines to regulate the TIME (182). Many studies showed that exosomes acted as bridging cytokines in the communication between HSCs and HCC cells.

**Figure 4:**
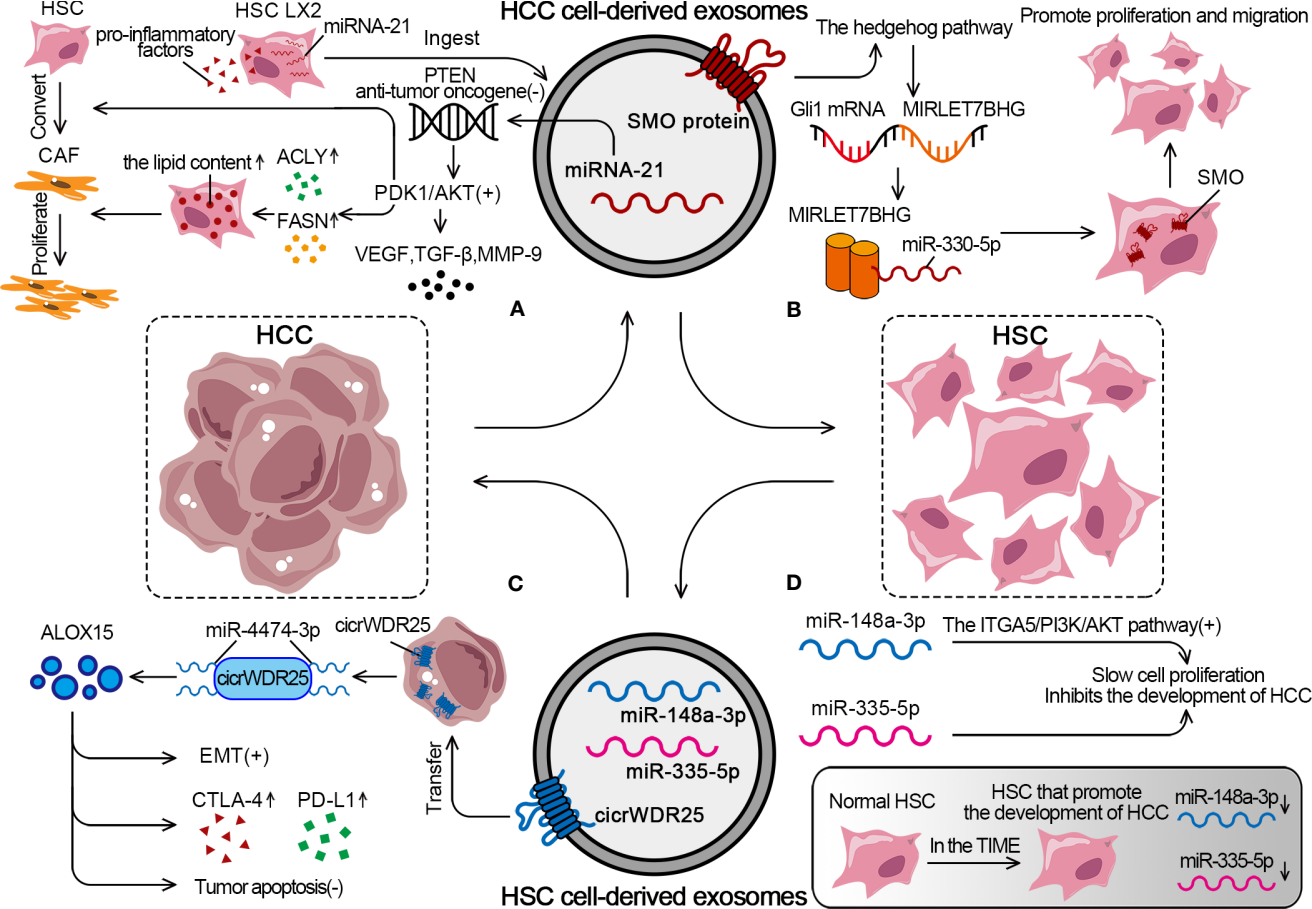
The detailed mechanism of exosomal effect between HSCs and HCC. **(A)** HCC cells can promote the conversion from HSCs to CAFs through exosomal miRNA-21. **(B)** HCC cells activate the SMO protein through exosomes to promote the proliferation and migration of HSCs. **(C)** HSCs derive exosomes to promote EMT, increase expression of CTLA-4 and PD-L1, inhibit tumor apoptosis. **(D)** In TIME, tumor suppressor miRs decreased, while rising in normal HSC.

Zhou demonstrated that HCC cells had a strong ability to convert HSCs into CAFs. The approach was to transfer miRNA-21 from HCC cells to HSCs in the tumor parenchyma via exosomes. MiRNA-21 converted HSCs into CAFs by downregulating its target PTEN antitumor oncogene and activating the PDK1/Akt signaling pathway (168). After ingesting HCC cell–derived exosomes, LX2 experienced an elevation in miRNA-21 expression, significant activation, and a notable increase in the expression of pro-inflammatory factors. Also, the activation of the Akt pathway upregulated the levels of adipogenesis-related enzymes ATP-citrate lyase and fatty acid synthase (FASN), leading to an increase in the lipid content in HSCs and promoting CAF proliferation (168, 183). Besides regulating lipid metabolism, the activation of the Akt pathway also promoted the release of pro-angiogenic substances (VEGF and TGF-β) and pro-tumor invasion substances (MMP-9) (**Figure 4A**). Xia found that HCC cells upregulated the expression of proto-oncogene smoothened (SMO), which is shared by both HCCs and HSCs, through the use of exosomes. The specific mechanism was that the SMO protein of HCC induced the hedgehog pathway to promote the expression of GLI family zinc finger 1 (Gli1) after being transduced to HSCs via exosomes. Gli1 mRNA acted as a transcription factor of microRNA let-7b host gene (MIRLET7BHG) and induced MIRLET7BHG transcription, which was the sponge for miR-330-5p to upregulate SMO expression in HSCs. SMO promoted EMT in HCC and cell proliferation and migration in HSCs (**Figure 4B**) (184).

As an essential component of the TIME, HSCs can regulate the behavior of HCC cells through the exosome pathway. Liu showed that HSC-derived exosomes transferred cicrWDR25 into HCC cells as a sponge for miR-4474-3p to upregulate the level of lipid-metabolizing enzyme arachidonate 15-Lipoxygenase (ALOX15). The final result was to promote the EMT in HCC and increase the expression of CTLA-4 and PD-L1. Notably, ALOX15 is associated with the inhibition of tumor apoptosis, and high peritumoral ALOX15 represents predict poor patient prognosis (**Figure 4C**) (185). HSCs in the TIME are converted from normal HSCs into HSCs that promote the development of HCC. MiR-148a-3p acts as a tumor suppressor and inhibits the development of HCC by activating the integrin subunit alpha 5 (ITGA5)/PI3K/Akt pathway. The overexpression of miR-148a-3p in HSCs significantly inhibits the proliferation of co-cultured HCC cells. However, miR-148a-3p expression is significantly downregulated in both HSCs and CAFs isolated from HCC tumor tissues, which causes a decrease in the number of miR-148a-3p-containing HSC-derived exosomes and promotes HCC (186). Similarly, miR-335-5p is the tumor suppressor that slows cell proliferation, promotes apoptosis, and limits cell invasion. It is also found to be significantly downregulated in HSCs in HCC. HSCs from the normal liver can secrete exosomes containing miR-335-5p to counteract the tumorigenic properties of HCC cells (**Figure 4D**) (187).

We have summarized the function of exosomes in the HCC innate immune microenvironment, adaptive immune microenvironment and immune microenvironment of other components in **Table 1**.

**Table 1:** The function of exosomes in the HCC immune microenvironment.

Source	Functional molecule (s)	Target cell/molecule	Response in the target cell/molecule	Reference
M1 macrophages	miR-628-5p	HCC	Suppress the promotion of HCC by inhibiting the expression of METTL14 and circFUT8	34
M1 macrophages	hsa_circ_0004658	HCC	Inhibit proliferation and promote apoptosis of HCC cells	35
HCC	miR-142-3p	M1 macrophages	Cause ferroptosis of M1 macrophages	38
HCC	miR-452-5p	Macrophages	Promote the polarization of M2 macrophages	41, 42
HCC	miR-146a-5p	Macrophages	Inhibit the expression of IFN-γ and promotes the polarization of M2 macrophages	39
HCC	LINC00662	Macrophages	Induce the polarization of M2 macrophages	45
HCC	lncRNA HMMR-AS1	Macrophages	Promote the polarization of M2 macrophages	46
HCC	hsa_circ_0074854	Macrophages	Foster the polarization of M2 macrophages and the EMT of HCC cells	27
M2 macrophages	miR-660-5p	HCC	Promote EMT in HCC cells	53
M2 macrophages	miR-27a-3p	HCC	Induce HCC cancer cell stemness and promotes tumor cell proliferation	50
M2 macrophages	miR-92a-2-5p	HCC	Inhibit the anti-tumor oncogene AR	58
M2 macrophages	MAAS	HCC	Promote the polarization of M2 macrophages and proliferation of HCC cells	59
M2 macrophages	lncMMPA	HCC	Increase the aerobic glycolysis in HCC cells	64
HCC	AFP,GPC3,HSP70	DCs	Stimulate of DC maturation and differentiation	68
HCC	dsDNA	DCs	Increase the expression of IFN-I and enhance DC-mediated cross-presentation of tumor antigens to cytotoxic T lymphocytes	69
HCC	lipid	DCs	Activate of mitochondrial fatty acid oxidation and induces DC immune dysfunction	73
DCs	AFP	HCC	Upregulate the expression of PD-L1 and SIRPα, increases the TGF-β,suppresses the proliferation of CD8+ T cells, induces Treg production	6
DCs	MHC-I and MHC-II	HCC	Promote the anti-tumor effects of T cells and NK cells	188
hCAF	IL-6	DCs	Impair the function of T cells and promotes Treg proliferation	74
Treg	miR-150-5p and miR-142-3p		Enhance inhibition of antitumor response and decreases T-cell activation	75
HCC	NKG2 ligand and TGFβ1	NK	Block the cell activation and impairs the function of NK cells	84
NK	circUHRF1	NK	Decrease the NK cell-derived IFN-γ and TNF-α and impairs NK cell function	90
NK	miR-186	HCC	Inhibit the proliferation of HCC cells	79
metastatic melanomas	PD-L1	CD8+T	Inhibit the function of CD8+T lymphocytes and promote tumor growth	106
HCC	miR-15a-5p	CD8+ T	Inhibit PD1 expression in CD8+ T cells and suppressed the development of HCC	116
colorectal cancer cell	circRHOBTB3	outside of cells	Excrete circRHOBTB3 out of cells to sustain cancer cell fitness	117
HCC	miR-146a-5p	Macrophages	Up-regulate the expression of Tim-3, LAG-3, CTLA4, TIGIT and PD-1 on T cell and downregulate the production of IFN- γ and TNF- α, induce T cell exhaustion	39
HCC	miR-23a-3p	Macrophages	Increase PD-L1 expression on macrophages and induce CD8+ T-cell apoptosis	8
HCC	circTMEM181	Macrophages	Damaging the function of CD8+T cells	120
M2 macrophage	miR-21-5p	CD8+T	Promote the exhaustion of CD8T cells	121
Treg		CD8+T	Decrease the secretion of perforin and IFN- γ from CD8+T cells	122
HCC	miR-155	CD4+T	Inhibit expression of PTEN and drives Th17 cell differentiation	103, 129
HCC	miR-21	CD4+T	Enhance Tregs proliferate and recruit to the TIME	133
HCC	miR-10b	CD4+T	Increasing Th1/Th17 and diminishing Th2/Treg cells	133
HCC	CircGSE1	CD4+T	Promote the differentiation of CD4+ T cells into Treg	136
HCC	14-3-3ζ	T cell	Impaire the functions, proliferation and activation of T cells and regulate their differentiation	101
HCC	HMGB1	TIM-1Breg cells	Expand TIM-1Breg cells,and inhibit the proliferation of CD8T cells and the production of TNF- α and IFN- γ	140
HCC	miR-1247-3p	fibroblasts	Convert NFs into CAFs	167
HCC	miR-21	HSC	Convert HSCs into CAFs	168
Hepatocytes (HBV infected)	miR-222	HSC	Inhibit ferroptosis in HSCs and upregulate the activation of HSC	177
Hepatocytes (HCV infected)	miR-1273g-3p	HSC	increase activation of HSC	178
Hepatocytes (fatty liver disease)	miR-1297	HSC	Increase the production of hydroxyproline, the activation marker α-SMA, and the proliferation marker PCNA	179
HSC (hepatitis)	miR-214	HSC	Promote expression of CCN2	180
HCC	miR-21	HSC	Enhance the conversion of HSC to CAF	168
HCC	SMO protein	HSC	Promote EMT	184
HSC	cicrWDR25	HCC	Promote EMT and increase the expression of CTLA-4 and PD-L1	185
CAFs	miR-92	Cancer cell	Promote apoptosis and impaired proliferation of T cells	169
CAFs	circZFR	HCC	Promote HCC resistance to cisplatin	172

## 5 Engineered exosomes

Engineered exosomes endow their cells and tissues with targeted specificity by modifying the surface molecules. Loading protein/RNA/small-molecule transporters into exosomes can achieve the purpose of treating diseased areas or cells (189). Exosomes produced by cells from different sources have different surface molecules anchored on their membranes, which gives them selectivity to specific receptor cells (190, 191). Meanwhile, exosomes, as endogenous functional biomolecules, have no toxicity and low immunogenicity, and exhibit biocompatibility, which provides conditions for selectively delivering drugs and genes to target cells and tissues (192, 193). According to these properties, exosomes of different sources have been put into research and clinical applications through various engineering design methods, such as engineered mesenchymal stem cells (MSCs) exosomes loaded with siRNA has been translated to the clinic (194, 195).

### 5.1 Designing of engineering exosomes

**Figure 5:**
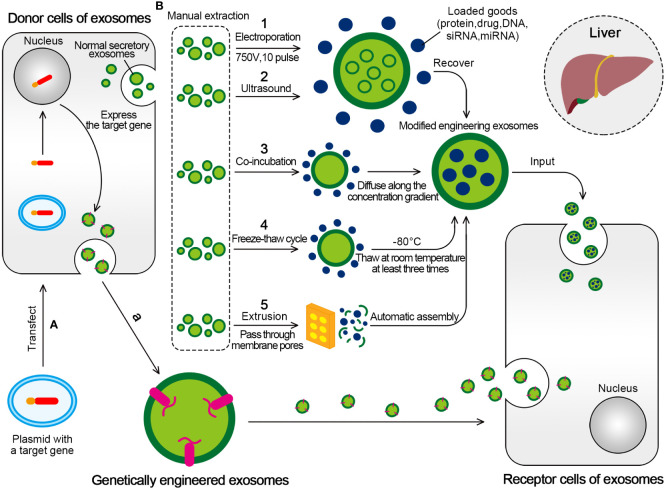
Two mainly transformation methods for engineered exosomes. **(A)** Active packaging, also known as genetic engineering. The donor cells that can produce exosomes can be transfected with plasmids with fusion genes, so that the exosomes produced by them have the target ligand. **(B)** Passive packaging, refers to embedding the targeted part into the exosome membrane through lipids or biological conjugation reactions. (1) Electroporation is formed by applying an external electric field (750V, 10 pulse) to exosomes to form hydrophilic pores, which allow hydrophilic molecules to load into exosomes, and eventually form engineered exosomes. (2) The ultrasonic treatment uses the mechanical shearing force of the ultrasonic probe to destroy the integrity of the exosome membrane and produce temporary pores, thus allowing the substances to be encapsulated for entry. (3) Drugs incubated with exosomes will produce exosomes containing drugs. The drugs interact with the lipid layer of the vesicle membrane and diffuse into the exosomes along the concentration gradient. (4) The drug was incubated with exosomes at room temperature for a fixed period, and the mixture was frozen together at –80°C, followed by thawing at room temperature at least three times. (5) Under physical stress, the membranes of liposomes and exosomes will rupture and reassemble drugs to form hybrid vesicles as they pass through membrane pores of controlled size.

The preparation methods for engineered exosomes are mainly divided into two categories (**Figure 5**). The first category is active packaging, also referred to as genetic engineering. This involves the packaging of the target transporter into exosomes through co-expression. The target ligand and the exosome membrane protein must be fused and inherited. Moreover, donor cells capable of producing exosomes can be transfected with plasmids containing fusion genes. This enables the exosomes produced by the donor cells to carry the target ligand (196, 197).

For example, the bioengineered cells Vero and Chinese hamster ovary cells can prepare exosomes carrying HBV-specific functional gRNA and Cas9 protein. These exosomes can deliver the gRNA and Cas9 protein to target cells. Hence, the delivery of the gene editing function in the clustered regularly interspaced short palindromic repeat (CRISPR)/Cas9 system is realized (198). To load the RAGE binding peptide (RBP) into exosomes, RBP was linked to the exosome membrane integration protein Lamp2b to generate RBP-linked exosomes (RBP-exo). The anti-inflammatory effects of RBP-exo were confirmed by cytokine assays in lipopolysaccharide (LPS)-activated macrophage cells (199).

The second category, passive packaging, refers to embedding the targeted part into the exosome membrane through lipids or biological conjugation reactions. The commonly used methods include electroporation (EP), co-incubation, extrusion, ultrasound, freeze-thaw cycle, and so on (196).

The EP is used to form hydrophilic pores by applying an external electric field. The destruction of the lipid bilayer of the membrane allows hydrophilic compounds, such as small DNA, miRNAs, and siRNAs, to be loaded into exosomes (200). EP is the most common method to encapsulate siRNAs or miRNAs into exosomes because of its simplicity, easy operation, and high loading efficiency (201).

Liang et al. loaded mir-26a into exosomes (derived from engineered human 293T cells) with an Apo-A1 sequence by EP. The apo-A1-modified exosomes bound specifically to HepG1 cells through SR-B2 receptors or were endocytosed by Hepg2 cells. Eventually, the cell cycle control proteins were downregulated, leading to the inhibition of cell migration and slowing down proliferation (202). In the study conducted by Mayela Mendt et al, the MSC-derived exosomes and freshly prepared human foreskin fibroblast (BJ fibroblast)–derived exosomes which were electroporated both inhibited oncogenic Kras expression in KPC689 tumors, substantially improved the histopathology of pancreas and exhibited a trending decrease in tumor weight and tumor burden at experimental endpoint, ultimately improved the overall survival of the mice (203).

Drugs are also delivered to exosomes by co-incubation, including drugs incubated with exosomes and drugs incubated with donor cells to produce exosomes containing drugs. Drugs interact with the lipid layer of the vesicle membrane and diffuse into the exosomes along the concentration gradient. The method is simple, but the loading capacity and efficiency are lower (204). Studies showed that paclitaxel (PTX) was incubated with mesenchymal stem cells to produce PTX-loaded exosomes, which showed significant antitumor effects on human pancreatic cells (CFPAC-1) *in vitro* (205). To increase anti-inflammatory effects, GyeungYun Kim et al. loaded curcumin into RBP-exo by incubation in phosphate-buffered saline (PBS) for 30 min at room temperature. Curcumin loaded RBP-exo (RBP-exo/Cur) had higher intracellular curcumin delivery efficiency than curcumin alone or curcumin loaded into unmodified exosomes (unmod-exo/Cur) (199).

The ultrasonic treatment uses the mechanical shearing force of the ultrasonic probe to destroy the integrity of the exosome membrane and produce temporary pores in the exosome membrane, thus allowing the substances to be encapsulated for entry and finally recovering the lipid bilayer (204).

For example, Lamichhane et al. sonicated the exosomes loaded with therapeutic siRNAs such that they were absorbed by the recipient cells and could target mRNA knockdown, resulting in decreased HER2 expression (206). After Kim et al. conducted an experiment where they incubated the exosomes at room temperature and observed that the amount of PTX loaded into the exosomes minimally increased. However, when they treated the exosomes with ultrasound, it resulted in the maximum loading capacity (207).

In the study conducted by Jiawei Zhao et al., 50 μg of dexamethasone(DEX)was mixed with 50 μg of exosomes at room temperature for 30 min followed by ultrasonic in a water bath sonicator (KQ-300DE). After ultrasonic, the mixture was incubated at 37°C for 1 h and further ultra-filtrated three times to remove excess free drugs. DEX-incorporated exosomes named Exo @ DEX, prepared by this method, was proved to effectively improve the accumulation of DEX in hepatic autoimmune hepatitis (208).

Freeze-thaw cycles were also used for the drug loading after exosome isolation. The drug was incubated with exosomes at room temperature for a fixed period, and the mixture was frozen together at –80°C, followed by thawing at room temperature at least three times. It had a mild effect and was suitable for loading miRNA and protein, but the drug loading was low (204, 209). Hamed Hajipour et al. used ultrasound and freeze-thaw cycles to load human chorionic gonadotropin (HCG) into the exosomes. When used as HCG-loaded exosomes, HCG had a significantly stronger effect on the upregulation of leukemia inhibitory factor and inhibition of Muc-16 expression compared with HCG or the exosome alone (210).

Under physical stress, the membranes of liposomes and exosomes rupture and reassemble drugs to form hybrid vesicles as they pass through membrane pores of controlled size. This method is called extrusion. Porphyrins were loaded into a triple-negative breast cancer cell (MD Anderson-Metastatic Breast-231)-derived exosomes in the Fuhrmann G assay. However, the exosomes formed by extrusion showed higher cytotoxicity than exosomes formed by other design methods (200).

### 5.2 Application and prospect of engineering in the immunotherapy of liver cancer

DEXs have great potential in the immunotherapy of HCC (188, 211). Lu et al. infected the DC cell line (DC2.4) established by transferring granulocyte-macrophage colony-stimulating factor (GM-CSF) (Csf2), Myc, and Raf genes into C57BL/6 mice with lentivirus-expressing murine α-fetoprotein (AFP) (6). It was found that these AFP-enriched DEXs could reshape the TIME of mice with HCC and trigger an effective specific antitumor immune response as a cell-free vaccine for the immunotherapy of HCC. In the study by Zuo et al., exosomes were used as the carrier of the HCC vaccine to initiate the specific immune response against HCC (212). Using the exosome anchor peptide method, they constructed an exosome decorated with HCC-targeting peptide (P47-P), AFP epitope (AFP212-A2), and the functional domain of high–mobility group nucleosome-binding protein 1 (HMGN1). This development paved the way for the individualized treatment of HCC immunity through the use of a universal exosome vaccine. Zhong et al. also demonstrated that the use of microwave ablation enhanced the antitumor effect of the DEX vaccine on HCC (213).

Tumor recognition can be enhanced by DC activation of TEXs carrying multiple tumor-associated antigens (214). Adding a powerful adjuvant and HMGN1 could enhance the ability of DCs to activate T cells and improve vaccine efficiency. Studies proved that TEXs coated with HMGN1 (TEX-N1ND) functional domain through the exosome anchor peptide enhanced the immunogenicity of DCs and triggered continuous antitumor immunity in large tumors in mice (215). At the same time, some studies showed that treating mice with ectopic and orthotopic HCC using TEX-pulsed DCs achieved significant tumor growth inhibition and promoted HCC-specific cytolysis (68). The exosomal lncRNA derived from tumor cells is also implicated as a signaling medium to coordinate cell function between adjacent tumor cells (216). Studies showed that HCC cell–derived exosomes contained high levels of lncRNA TUC339, which regulated the production and phagocytosis of macrophage cytokines as well as M1-type polarization in M2-type transformation. Therefore, the engineering design of tumor-derived exosomal lncRNA has become a hot research topic recently; it also reveals the complex interaction between tumor cells and innate immunity (216).

The treatment based on miRNAs has great application prospects in clinical antitumor treatment, but it lacks a good carrier for delivering miRNA drugs. MSC-derived exosomes are an important topic in cancer treatment and are considered a double-edged sword (217). Whether MSC-derived exosomes are involved in immunosuppression or immune promotion in the TIME is controversial, the uncertainty of its function or the harboring of harmful contents and other factors leads to its uncertain biosafety in clinic (218). However, MSC-derived exosomes hold remarkable potential in cancer immunotherapy applications, have been proved to be safe in many studies, and thus represent an excellent drug delivery carrier (219). Mahati et al. designed a nano-delivery system using exosomes modified with a single-stranded variable fragment (scFv) from human umbilical cord blood MSCs (219). The exosomes modified with anti-GPC3 scFv were obtained by genetic engineering technology, and then the miR-26a simulants were loaded by EP. The results showed that the anti-GPC3scFv-modified exosomes inhibited the proliferation and migration of GPC3-positive HCC cells by regulating the expression of the downstream target genes of miR-26a. Meanwhile, adult MSCs have been proved to have the perfect tumor-homing ability, and the microRNA encapsulated by MSC-derived exosomes plays a role in intercellular communication (220). In view of this characteristic, MSCs designed to secrete miR-379-rich exosomes have been used for the *in vivo* treatment of liver and breast cancer (220). Some studies also found that miR-15a-rich MSC-derived exosomes retarded HCC development via the downregulation of SALL4 (221). Similarly, miR-148a-rich MSC-derived exosomes protected against liver fibrosis by targeting the Kruppel-like factor 6 (KLF6)/STAT3 pathway in macrophages (222). Recent studies found that MSC-derived exosomes were engineered to carry siRNA or antisense oligonucleotide (ASO) targeting STAT3 (iExosiRNA-STAT3 or iExomASO-STAT3) they have significant anti-liver fibrosis effects and have been translated to the clinic (195).

Studies have shown that exosomes isolated from MSCs derived from bone marrow (BMSCs) can protect hepatocytes from apoptosis and improve the survival rate of D-galactosamine/TNF-α-induced acute liver failure in mice (223). Meanwhile, studies have also found that norcantharidin (NCTD, an anti-cancer drug) can be loaded into BMSCs-derived exosomes by EP, which enhances the treatment of HCC (224). Kenji Takahashi et al. found that miR-122-transfected adipose tissue–derived MSCs (AMSCs) could effectively package miR-122 as a secreted exosome, which improved the sensitivity of cancer cells to chemotherapy by improving the expression of miR-122 target genes in HCC cells (225). Lou et al. also found that miR-199a-modified AMSCs-derived exosomes(AMSC-Exo-199a) can improve HCC chemosensitivity (226). Another study found that RNAi-mediated knockdown of exosomal lncRNA reduced the function and progression of tumor cells in HCC and thus promoted the treatment of HCC via mediating the chemotherapeutic stress response (227). Wang et al. found that astrocyte-derived exosomes could carry miR-335-5p. The miR-335-5p could be introduced into hepatoma cells to inhibit tumor growth and metastasis, thus providing a new therapeutic strategy for HCC (228). Recently, the gene editing of Cas9 ribonucleoprotein (RNP) has attracted increasing attention. Some studies reported a new genome editing and delivery system, which was called exosome^RNP^, where Cas9 RNP could be loaded into purified exosomes isolated from HSCs. This exosome^RNP^ could upregulate cyclin E1, apoptosis, and K (lysine) acetyltransferase 5 by targeting p53, which showed strong therapeutic potential in acute liver injury, chronic liver fibrosis, and HCC mouse models (229).

Belhadj et al. proposed a combined “eat me/don’t eat me” strategy to avoid the phagocytosis of macrophages by modifying macrophages with CD47-enriched exosomes (230). Du et al. further verified the effectiveness of this combined strategy. They performed surface functionalization of the exosome with CD47, loading the membrane with a ferroptosis inducer (Erastin, Er) and filling the core with a photosensitizer (Rose Bengal, RB). These drug-loaded exosomes (Er/RB@ExosCD47) significantly induced ferroptosis in tumor cells (231). Some studies reported a unique method based on exosomes and TAMs, reprogramming TAMs to the pro-inflammatory M1 phenotype by selectively delivering STAT6-targeted ASO to TAMs. This new engineered exoASO-STAT6 showed strong antitumor activity as a single therapy in many preclinical tumor models (232). Lee et al. also found that M1 macrophage-derived exosomes were transfected with NF-κB p50 siRNA and miR-511-3p and were surface-modified with IL4RPep-1, an IL4R-binding peptide, to target the IL4 receptor of TAMs (IL4R-Exo(si/mi), which inhibits tumor growth by reprogramming TAMs to pro-inflammatory M1 phenotype (233). As discussed earlier, NK-exo plays a key role in enhancing the tumor-targeting and antitumor activity in HCC *in vitro* and *in vivo*, so the bioengineered design based on NK-exo may help improve the therapeutic effect of NK-exo and its antitumor effect (234). However, the bioengineered design of specific NK-exo still needs further investigation.

Engineered exosomes can also play a significant synergistic role in treating HCC. Sorafenib is one of the few effective first-line agents approved for treating advanced HCC. Shi et al. showed that a combination of DC-TEX and PD-1 antibody (Ab) enhanced the efficacy of sorafenib (235). Li et al. found that siGRP78-modified BMSCs-derived exosomes can inhibit Sorafenib resistance in HCC (236). Studies proved that sorafenib enhanced anticancer activity mainly by inducing ferroptosis. They also found that HCC-targeted exosomes (ExoSP94-Lamp2b-RRM) could enhance sorafenib-induced ferroptosis by inhibiting the expression of glutathione peroxidase 4 (GPX4) and dihydroorotate dehydrogenase (DHODH). This increased the sensitivity of HCC to sorafenib, providing new ideas for the clinical application of sorafenib from the perspective of ferroptosis (237).

Furthermore, recent studies have suggested a potential relationship between ferroptosis and the TIME and immune cells (238). The polarization of M2-type TAMs in the TIME to M1-type TAMs is a commonly employed strategy in the realm of cancer immunotherapy. However, Hsieh et al. found that zero-valent-iron nanoparticles induced the ferroptosis of cancer cells like ferroptosis inducers and efficiently repolarized macrophages from M2 to M1 phenotype (239). Although some studies have shown that many nanoparticles can participate in regulating the TIME, direct evidence regarding the regulation of TIME by ferroptosis inducers is still lacking. However, this also reminds us that we can regulate the TIME and related immune cells by combining engineered exosomes with ferroptosis, thus creating a novel foundation for TIME and immunotherapy.

Meanwhile, we have summarized the engineered exosomes in **Table 2**.

**Table 2:** The functional molecules in engineering exosomes and their source cells.

Source	Components	Functions	Reference
DC (DEX)	AFP,GM-CSF (Csf2),Myc,Raf ,C57BL/6 mice (DC2.4)	Reshape the TIME of HCC mice and trigger an effective specific anti-tumor immune response	6
DC (DEX)	microwave ablation	Enhance the anti-tumor effect of DEX vaccine on HCC	213
TEX	HMGN1(TEX-N1ND)	Enhance the immunogenicity of DC and trigger continuous anti-tumor immunity in large tumors of mice	215
TEX	TEX-pulsed DCs	Achieve significant tumor growth inhibition and promote HCC-specific cytolysis	68
DC-TEX	DC-TEX and PD-1 antibody (Ab)	Enhance the efficacy of sorafenib	235
HCC	P47-P,AFP212-A2 and N1ND-N	The universal exosome vaccine for HCC immunity	212
HCC	lncRNA TUC339	Regulate the production and phagocytosis of macrophage cytokines as well as M1-type polarization in M2-type transformation	216
HCC	ExoSP94-Lamp2b-RRM,multi-siRNA	Increase the sensitivity of HCC to sorafenib	237
MSCs	anti-Glypican3 (GPC3) scFv,miR-26a	Inhibit proliferation and migration of GPC3-positive HCC cells	219
MSCs	miR-379	Use for *in vivo* treatment of liver cancer	220
MSCs	miR-15a	Retard HCC development	221
MSCs	miR-148a	Protect against liver fibrosis	222
MSCs	siRNA,antisense oligonucleotide (ASO),STAT3	Have significant anti-liver fibrosis effects	195
MSCs	Norcantharidin (NCTD)	Enhances the treatment of HCC	224
MSCs	miR-122(AMSCs)	Improve the sensitivity of cancer cells to chemotherapy	225

## 6 Conclusion and future prospects

In recent years, more and more evidences indicated exosomes play an important role in tumor progression as nanomolecules for intercellular communication. TIME is the environment in which tumor cells rely for survival. Numerous studies have shown that exosomes play a very important role in intercellular communication. Understanding the mechanism of cell interaction between different TIMEs is of great significance for cancer treatment and the research progress of engineered exosomes. In this review, we summarized five cell components that are very important in the HCC immune microenvironment, and described in detail the role of exosomes in different cell communication in cancer progression. In addition, we list the engineering exosomes that have been used in emerging drug delivery systems in recent years. Engineered exosomes have been proved to have good clinical application potential in the field of immunotherapy of HCC, and show better therapeutic effects, low immunogenicity and targeting than nanoparticles. However, due to restrictions for various reasons, such as low biological safety and drug loading efficiency, the clinical transformation of engineered exosomes still faces many problems and challenges. Although our review provides examples of engineered exosomes for clinical transformation, it is still relatively lacking and more research is needed to confirm their clinical application.

## Author contributions

TL, JJ, HK, and WO all contributed to the manuscript writing. TL and LW contributed to the figure production. HK contributed to the table production. XL and PJ reviewed and modified the manuscript. All authors contributed to the article and approved the submitted version.

## Glossary

14-3-3 ζ: 14-3-3 protein zeta
ACLY: ATP-citrate lyase
AFP: α
ALDH1A3: Aldehyde Dehydrogenase 1 Family Member A3
ALOX15: Arachidonate 15-Lipoxygenase
AMSCs: Adipose tissue-derived mesenchymal stem cells
AR: Androgen receptor
ARID3A: AT-Rich Interaction Domain 3A
B4GALT3: Beta-1,4-Galactosyltransferase 3
Bregs: regulatory B-cells
CAFs: Cancer associated fibroblasts
CALM1: Calmodulin 1
CCL2: Chemokine (C-C motif) ligand 2
CCN2: Connective tissue growth factor
CcnE1: cyclin E1
CD: Cluster of Differentiation
ceRNA: competitive endogenous RNA
cGAS/STING pathway: GMP-AMP synthase/stimulator of interferon genes pathway
Chi3L1: chitinase 3-like 1
CHO: Chinese hamster ovary
circUHRF1: Circular Ubiquitin-like, containing PHD and RING finger domains, 1
CLCF1: Cardiotrophin-like cytokine factor 1
CRISPR/Cas9 system: clustered regularly interspaced short palindromic repeat./associated nuclease 9 system
CSF-1: Colony-stimulating factor 1
CTL: Cytotoxic lymphocytes
CTLA4: Cytotoxic T-lymphocyte-associated protein 4
CXCL1: Chemokine (C-X-C motif) Ligand 1
DCs: Dendritic cells
DDP: Cisplatin
DEX: Dexamethasone DEXs, DC-derived exosomes
DPP4: Dipeptidyl peptidase-4
ECM: Extracellular matrix
EMT: Epithelial-mesenchymal transition
EP: Electroporation
ER: Endoplasmic reticulum
Er: Erastin
EVs: Extracellular vesicles
FAO: Fatty acid oxidation
FASN: Fatty Acid Synthase
FBXO16: F-box protein 16
FXR: Farnesoid X receptor
GATA3: GATA Binding Protein 3
Gli1: GLI family zinc finger 1
GLUT1: glucose transporter protein 1
GM-CSF: granulocyte-macrophage colony-stimulating factor
GOLM1: Golgi membrane protein 1
GPC3: Glypican 3
GRP78: Glucose-Regulated Protein 78
HBX: Hepatitis B virus X protein
hCAFs: HCC-associated fibroblasts
HCC: Hepatocellular carcinomas
HCV: Hepatitis C virus
HepG2: Human hepatocellular carcinomas 2
HIF-1α: Hypoxia-inducible factor -1α
HK2: Hexokinase 2
HMGB1: High Mobility Group Protein 1
HMMR-AS1: hyaluronan mediated motility receptor- antisense RNA 1
HSCs: Hepatic stellate cells
HSP70: Heat shock protein 70
HuR: Human antigen R
ICAM-1: Intercellular cell adhesion molecule-1
IDO: Indoleamine 2,3-dioxygenase
IFI30: Interferon γ-inducible protein 30
IFN-γ: Interferon-γ
IL: Interleukin
JAM3: Junctional Adhesion Molecule 3
KAT5: K (lysine) acetyltransferase 5
KLF3: Kruppel Like Factor 3
LAG-3: Lymphocyte-activation gene 3
LPS: Lipopolysaccharide
MAAS: MAPKAPK5-AS1
MHC I: Major histocompatibility complex class I
MICA: The major histocompatibility complex class I polypeptide-related sequence A
MMPs: Matrix metalloproteinases
MORC3: MORC family CW-type zinc finger 3
MSCs: mesenchymal stem cells
N1ND-N: nucleosome-binding protein 1
NCTD: Norcantharidin NFs, Normal fibroblasts
NF-κB: Nuclear factor kappa-B
NK cells: Natural killer cells
NKG2D: Natural killer group 2D
NorCA: Norcholic acid
NP: Nanoparticles
OCT-4: Octamer-binding transcription factor-4
p-AKT: Phospho-phosphatidylinositol 3-kinase-protein kinase B
PBS: phosphate-buffered saline
PCED1B-AS1: PC-Esterase Domain Containing 1B-antisense RNA 1
PCNA: Proliferating Cell Nuclear Antigen
PD-1: Programmed death-1
PDK1/AKT: Phosphoinositide-dependent kinase-1/protein kinases
PD-L1: Programmed death-ligand 1
PGE2: Prostaglandin E2
PPARα: Peroxisome proliferator activated receptor α
PTEN: Phosphatase and tensin homolog
PTX: Paclitaxel
PUMA: p53 upregulated modulator of apoptosis
RAGE: Receptor for advanced glycation end products
RB: Rose Bengal
RBP: RAGE binding peptide
RBPJ: Recombination signal binding protein-Jκ
RNP: Ribonucleoprotein
SALL4: Sal-like protein-4
cFv: Single-stranded variable fragment
SHP: Small heterodimer partner
SIRPα: Signal-regulated protein α
SLC3A2: Solute carrier family 3 member 2
SMO: Smoothened
SOCS1: Suppressor of cytokine signaling 1
SOX2: SRY-Box Transcription Factor 2
STAT: Activate transcriptional activator
TAMs: Tumor-associated macrophages
TEXs: Tumor-derived exosomes
TFRC: Transferrin receptor
TGF-β: Transforming growth factor β
TH2: T helper 2
HMGB1-TLR2/4-MAPK pathway: High Mobility Group Protein 1-Toll like receptor 2/4 and mitogen-activated protein kinase signaling pathways
THP-1: Human monocytic-leukemia cells
TIGIT: T cell immunoreceptor with Ig and ITIM domains
Tim-3: T cell immunoglobulin and mucin domain-containing protein 3
TIMP3: Tissue inhibitor of metalloproteinases-3
TMIE: Tumor immune microenvironment
TNF-α: Tumor necrosis factor alpha
Tregs: Regulatory T lymphocytes
TXNIP: Thioredoxin Interacting Protein
ULBP1–6: UL16-binding proteins
VEGF: Vascular endothelial growth factor
WNT3A: Wnt Family Member 3A
XPO1: Exportin 1
YAP1: Yes-associated Protein 1
ZVI: Zero-valent-iron
α-SMA: α-smooth muscle actin.
